# Geographical Inequalities in the Distribution of Healthcare Specialists in Poland

**DOI:** 10.3390/healthcare14152427

**Published:** 2026-08-06

**Authors:** Justyna Rój

**Affiliations:** Department of Operational Research and Mathematical Economics, The Poznań University of Economics and Business, Al. Niepodległości 10, 61-875 Poznań, Poland; justyna.roj@ue.poznan.pl

**Keywords:** healthcare, specialists, inequality

## Abstract

**Background/Objectives**: Healthcare specialists are fundamental to the healthcare system, so ensuring their fair distribution is essential for providing sustainable, equitable services. This study aimed to evaluate geographical inequity in the distribution of healthcare specialists across Poland’s macro-regions from 2018 to 2024. **Methods**: Data were obtained from Statistics Poland. Healthcare specialist availability was measured as density rates per 10,000 population and per km^2^. Population- and geographic-based inequalities were assessed using the unitless Gini coefficient (0–1), and the unitless Theil index. **Results:** The analysis yielded several key findings. Firstly, the spatial distribution of healthcare specialists is considerably less equitable than their distribution relative to population size, with clear differences between macro-regions. Access to oncologists and cardiologists was particularly unequal. These patterns demonstrate persistent structural imbalances in specialist availability. **Conclusions:** The results of this study can inform more effective policies aimed at achieving a fairer distribution of specialist care across the country. Other countries characterized by similar issues can build on these findings and adapt them to their own contexts.

## 1. Introduction

This research examines geographical inequities in the distribution of healthcare specialists in Poland. Ensuring the right to the highest attainable standard of health—and, consequently, universal access to care and effective service coverage—requires not only sufficient supply of healthcare human resources but also their equitable distribution, accessibility, competence, motivation and support from the wider healthcare system [1]. The consequences of an inequitable distribution of the healthcare workforce are substantial. Geographical inequalities in the distribution of healthcare professionals can restrict access to services and contribute to poorer health outcomes, particularly in rural and socioeconomically disadvantaged regions [2,3]. Such inequities may undermine equal opportunities to attain good health. Therefore, ensuring equity in healthcare is a fundamental principle, as it guarantees that all individuals have an equal opportunity to achieve their full health potential, regardless of their demographic, social, geographic, or economic circumstances [4,5].

Equitable distribution of the healthcare workforce is not only a priority for healthcare systems and also an important component of sustainable development. The Sustainable Development Goals [6] and the World Health Organization [7] emphasize that achieving universal health coverage requires healthcare systems supported by a skilled and equitably distributed workforce, ensuring access to high-quality healthcare services for the entire population [7,8,9,10,11].

In line with these global priorities, many international initiatives [12,13,14,15,16] address the need for a more equitable distribution of the healthcare workforce. One such initiative is the WHO Global Strategy on Human Resources for Health: Workforce 2030 [1], which identifies reducing inequities in the distribution of healthcare workers as a key milestone to be achieved by 2030. Accordingly, many European and national policies aim to develop and sustain a skilled workforce capable of meeting increasingly complex health needs [17,18].

At the same time, demographic, epidemiological, and systemic changes are reshaping health needs globally. The aging population, the rising prevalence of chronic conditions, and the evolving disease patterns are increasing the demand for specialized healthcare services [19,20], placing unprecedented pressure on healthcare specialists, particularly in fields such as geriatrics, psychiatry, and oncology [21]. This puts a strain on solidarity-based healthcare systems operating under resource constraints [20]. These demographic and epidemiological changes increase the demand for specialized healthcare services, making the equitable geographical distribution of healthcare professionals increasingly important. Furthermore, disasters—whether natural or human-made—can further disrupt healthcare delivery by damaging infrastructure and reducing workforce capacity. This was tragically demonstrated during the COVID-19 pandemic through the loss of healthcare workers themselves. The pandemic further intensified pressures on healthcare systems, revealing vulnerabilities in staffing, especially critical gaps in specialized areas [19].

In response to these pressures, healthcare systems must optimize and standardize human resources to remain resilient [21]. This requires proactive strategies that align with changing population needs and are supported by long-term investment in healthcare human resources [1]. Although, education and training are central to shaping workforce capacity [22], their long duration means the immediate supply depends on existing staff, and new investments only have long-term effects [23]. Therefore, balancing current pressures with future needs is therefore a major challenge. Rising demand and limited resources underscore the importance of precise and forward-looking planning that—according to the European Commission [24]—must account for multiple external and internal determinants such as population aging, changing health needs, migration, technological innovation and workforce aging, recruitment and retention, skills mismatches and geographic distribution.

Within this framework, improving the equitable geographic distribution of healthcare specialist is crucial because it directly determines the availability and quality of healthcare services [25,26,27]. While a perfectly equitable distribution is utopian, strategies informed by a solid understanding of workforce dynamics can still lead to significant improvements [28].

Despite its critical importance, global investment in the health workforce remains insufficient [29]. There are shortages in both quantity and geography, with significant challenges in staffing rural and underserved areas. A growing mismatch between workforce competencies and population health needs has also been documented [19,21].

A substantial body of international literature documents persistent geographic inequities in the distribution of the healthcare workforce across countries. A synthetic review of these studies was presented by Rój, 2020 [18], and more recent studies continue to confirm these findings. Such inequalities have also been observed in other countries, among others in China, where analyses were conducted from 2010 to 2021 [30,31,32], as well as in Iran [33,34], Turkey [35], Italy [36], England [37], Hungary [38], Finland [39], and other European countries [40]. These studies show that intra-regional disparities are a major contributor to overall inequity, often exacerbated by allocation policies that prioritize population size over geographic barriers [32]. As a result, healthcare professionals tend to cluster in urban, economically developed regions, leaving rural and less developed areas underserved [41].

Also, recent evidence shows that theses regional differences were further by the COVID-19 pandemic due to uneven economic and social impacts [42,43]. Therefore, monitoring the distribution of healthcare specialists is essential for evidence-informed regional workforce planning and targeted policy interventions by identifying the medical specialties and geographical areas at the greatest risk of limited access to specialist services.

Poland represents a particularly relevant case for such analysis. Although equal access to healthcare is guaranteed by the Constitution and supported by the National Health Fund (NFH—The National Health Fund (Narodowy Fundusz Zdrowia, NFZ) is Poland’s single public health insurance institution responsible for financing and contracting healthcare services for insured residents—more in [18]) the country continues to experience shortages of medical personnel and considerable regional variation in healthcare workforce distribution [18,44], and remains among the most understaffed in Europe, with only 3.9 doctors per 1000 population, which is at the average of OECD 37 [44]. These challenges highlight the importance of regularly assessing how healthcare specialists are distributed across regions.

The aim of this study is to examine the geographical distribution of major groups of healthcare specialist in Poland and to assess regional inequalities in their distribution relative to area and population size. More specifically, the study investigates whether the geographical distribution of healthcare specialists differs across professional groups and whether they vary depending on whether distribution is assessed relative to population or area size.

Based on previous evidence documenting persistent regional inequalities in Poland [18], this study hypothesizes that substantial geographical inequalities in the distribution of healthcare specialists persist over the study period and vary across specialist groups. This study extends previous research [18] and fills a significant gap in the Polish context [45,46,47]. By providing evidence for the period 2018–2024 and examining inequalities both between and within Polish macro-regions, as well as using population- and area-based indicators, this study contributes to the literature on healthcare workforce distribution. The study also provides evidence to inform regional workforce planning and health policy by identifying the healthcare specialties and macro-regions at the highest risk of geographical inequities in specialist distribution and such evidence can support targeted policy interventions by helping policymakers prioritize workforce allocation, residency planning, NHF contracting, recruitment and retention strategies, and investments in regional training capacity.

This article is organized as follows. After the introduction (Section 1), the methodology is presented in Section 2. Section 3 outlines the results, which is followed by broader discussion in Section 4 and conclusions in Section 5.

## 2. Materials and Methods

### 2.1. Materials

Poland, the largest EU Member State among those that acceded after 2004, with a population of approximately 37.5 million and a total area of 312,685 km^2^ in 2025 [48], was selected as the case study for two reasons. First, this study builds on and extends previous analyses conducted for an earlier time period by [18], making Poland a logical setting for maintaining both methodological and temporal continuity. This consistency enables a meaningful examination of long-term trends and shifts in the geographical distribution of healthcare specialists. Second, Poland is known for persistent structural challenges, including clear regional differences in access to medical services, an aging population, and a shortage of medical staff. There are also significant disparities in the distribution of specialists across voivodeships [18]. These characteristics make the Polish healthcare system a particularly suitable context for investigating equity in the geographical distribution of the workforce, especially since in 2018, the leading causes of death in Poland were diseases of the circulatory system (approximately 41%), cancer-related mortality (around 25%), and respiratory diseases (6–7%). These figures reflect long-standing public health challenges, particularly the high burden of cardiovascular conditions among older adults. By 2023, the overall structure of mortality remained largely unchanged, with these three categories still accounting for the majority of deaths. However, there was a slight decline in the proportion of deaths due to circulatory diseases, dropping to approximately 37%, suggesting gradual improvements in cardiovascular prevention and treatment. Meanwhile, cancer-related mortality increased modestly to 27%, and respiratory diseases rose to 7.4%, particularly among women. These trends underscore the persistent dominance of chronic non-communicable diseases in Poland’s mortality profile, while also highlighting emerging concerns related to respiratory and mental health conditions [49].

The analysis covered the period 2018–2024, with 2024 being the most recent year for which complete data had been officially published by Statistics Poland (GUS) at the time of the study. Data on healthcare specialists were obtained from the Knowledge Database Health and Healthcare of Statistic Poland for the years 2018–2024 [50], an official statistical database maintained by Statistics Poland that provides regularly updated health-related statistics and metadata. The database publishes information for 16 specialist categories, (and since 2021, 18 specialist categories) which determined the scope of the analysis. Although the analysis is limited to specialties for which consistent official data are available, the included groups cover the majority of the specialist workforce in Poland (representing almost 70% of all specialists in Poland).

Data on population and area size were obtained from the Statistical Yearbooks of Statistics Poland [48], which constitute the official source of demographic and territorial statistics in Poland, published by Statistics Poland (GUS). The number of specialists was recalculated per 10,000 inhabitants and per square kilometer to assess workforce density. Healthcare specialist density was calculated per 10,000 inhabitants and per square kilometer to assess the distribution of healthcare specialists relative to size of population and land area.

The primary territorial unit of analysis was the voivodeship (N = 16), which represents the highest level of Poland’s administrative division, as this was the lowest geographical level for which consistent data were available. According to the Nomenclature of Territorial Units for Statistics (NUTS), a hierarchical classification system developed by Eurostat to facilitate the comparability of regional statistics across European countries, Poland has been divided into seven macro-regions (NUTS 1), 17 regions (NUTS 2), and 73 sub-regions (NUTS 3) since 1 January 2018.

The NUTS classification does not fully correspond to Poland’s administrative division. Fifteen voivodeships (Lower Silesian, Kuyavian-Pomeranian, Lubelskie, Lubuskie, Łódzkie, Lesser Poland, Opolskie, Podkarpackie, Podlaskie, Pomeranian, Silesian, Świętokrzyskie, Warmian-Masurian, Wielkopolskie, and West Pomeranian) each correspond directly to a single NUTS 2 region. The sixteenth voivodeship, Mazowieckie, is divided into two NUTS 2 regions: Warszawski Stołeczny and Mazowiecki Regionalny. Because separate statistical data for these two NUTS 2 regions were not available, they were analyzed jointly as a single territorial unit corresponding to the Mazowieckie Voivodeship. Consequently, all analyses at the regional level used 16 voivodeships.

To examine geographical inequalities at different spatial scales, the 16 voivodeships were subsequently assigned to the seven NUTS 1 macro-regions (central, northwestern, southwestern, northern, southern, eastern, and Mazovian). This enabled the assessment of inequalities both between and within macro-regions, as well as the decomposition of overall inequality into between- and within-macro-region components.

However, the study period was affected by methodological change: starting in 2021, healthcare specialist data has been collected using a revised methodology by the official statistics institution, which provides a more accurate estimate of the medical workforce. Therefore, the interpretation of the results required a cautious approach and separate analyses of the two study periods.

### 2.2. Methods

To evaluate the geographical distribution of healthcare resources, the study applied the Gini coefficient calculated using the Lorenz curve. These methods are widely used in research on inequalities of geographical distribution as they provide a concise and robust measure of disproportions in resource allocation. This methodological framework also allows for comparison with the results of earlier research (Rój, 2020) [18], which employed the same analytical approach and where these methods were also widely discussed and presented.

The Gini coefficient, originally developed by Corrado Gini [51] as a measure of inequality is calculated using the Lorenz curve and reflects the ratio of the area between the curve and the line of perfect equality to the total area under that line. This method illustrates the cumulative share of resources in relation to the cumulative share of the population and reflects the degree of deviation from perfect equality. Smaller deviations from the line of equality indicate a more even distribution.

The Gini coefficient ranges from 0 to 1 [52,53], where 0 indicates perfect equality (uniform distribution of specialists relative to population or area), while 1 indicates complete inequality (all specialists concentrated in a single region). Following the interpretation adopted based on the approaches in previous studies [54], Gini coefficient values below 0.30 were interpreted as indicating a desirable level of equality, values between 0.30 and 0.40 as an acceptable but uneven distribution, values from 0.40 to 0.50 as reflecting high inequality, and values above 0.50 as indicating a highly inequitable distribution.

Two measures were used to assess inequality: one describing how the number of healthcare specialists relates to the size of the population, and then to area size. The Gini coefficient was then derived using the following formula [52,53]:

(1) $$G\left(y\right)=\frac{\sum_{i=1}^{n}\left(2i\;-\;n\;-\;1\right)y_{i}}{n^{2}\bar{y}}$$

where

*y_i_*—the value of i-observation

n—denotes the total number of observations

To further identify the factors contributing to inequality, the Theil index was applied. This measure also captures inequity, but unlike the Gini coefficient, it enables the decomposition of overall inequality into contributions from smaller subgroups within a larger system [55]. In this study, the Theil index was used both to assess the general level of equity in the distribution of healthcare specialists in Poland and to determine the share of inequality attributable to each macro-region. The index ranges from 0 (complete equality) to 1 (maximum inequality) and is calculated as follows:

(2) $$Theil\;index=\;\sum_{i=1}^{n}P_{i}\cdot ln\left(\frac{P_{i}}{Y_{i}}\right)$$

where

*P_i_*—the share of the population in a given region or macro-region relative to the total population;

*Y_i_*—the share of healthcare specialists in that region or macro-region relative to the overall healthcare specialists.

If the study area is divided into several groups (e.g., k groups in the formula such as the central, northwestern, southwestern, northern, southeastern and Masovian regions in this case), the Theil index can be decomposed into two components into within the contributions rates—the *T_intra_*_−*class*_ and between groups contribution rates—*T_inter_*_−*class*_.

By expressing the overall Theil index as the share of *T_intra_*_−*class*_ and *T_inter_*_−*class*_, it becomes possible to determine how much of the observed inequality originates within individual macro-regions and how much results from differences between them. A higher proportion of *T_intra_*_−*class*_ points out that inequity in the distribution of healthcare specialists arise mainly from variations in their density inside the groups (macro-regions). Conversely, a higher proportion of *T_inter−class_* shows that inequality is driven primarily from the variations in healthcare staff density across groups, meaning between macro-regions such as central, northwestern, southwestern, northern, southern and eastern Poland, Masovian and vice versa [56,57].

*Theil index = T_intra−class_ + T_inter−class_*

(3) $$T_{intra-class}=\sum_{g=1}^{k}P_{g}\cdot T_{g}$$

$$T_{inter-class}=\sum_{g=1}^{k}P_{g}ln\left(\frac{P_{g}}{Y_{g}}\right)$$

*T_intra_*_−*class*_: degree of equity in healthcare specialists’ distribution within the macro-regions.

*T_inter_*_−*class*_: degree of equity in healthcare specialists’ distribution between the macro-regions.

*T_g_*: Theil index of healthcare specialists’ distribution in macro-region.

*P_g_*: proportion of population in one macro-region accounting for the total population.

*Y_g_*: proportion of healthcare specialists in one macro-region accounting for the total healthcare specialists [58,59].

All analysis was performed in Microsoft Excel for Microsoft 365.

## 3. Results

Analysis of the specialty structure showed that it remained relatively stable throughout the study period, with only minor changes following the 2021 revision of the reporting methodology (Supplementary Materials). Internal medicine, surgery, and family medicine consistently accounted for the largest share of specialists (approximately 44–50%), followed by pediatrics, anesthesiology and intensive therapy, obstetrics and gynecology, and cardiology. The remaining specialties each represented less than 5% of the specialist workforce. The marked increase in the share of internists from 2021 onwards most likely reflects the methodological change rather than a true increase in workforce capacity.

The results showed that the density of various groups of healthcare specialists expressed as number of per 10,000 population increased from 2018 to 2024. However, the magnitude of these changes varied across the analyzed groups of specialists (Table 1), yet still indicated improved availability relative to the population size.

The analysis of the entire study period should be interpreted with caution due to a change in the methodology used to calculate the number of specialists from 2021 onwards. Therefore, the periods 2018–2020 and 2021–2024 were analyzed separately. The number of specialists per 10,000 population increased in all analyzed specialties, except for pediatricians, for whom a slight decrease was observed between 2018 and 2020. However, in both analyzed periods, the largest relative increases were recorded among oncologists, psychiatrists, and cardiologists. In addition, during 2021–2024, substantial increases (7% or more) were observed not only in these specialties but also among radiologists, family medicine physicians and endocrinologists.

Before the methodological change in 2020, the largest specialty groups (defined as more than 1.5 specialists per 10,000 population) were surgeons, family medicine physicians, and internists. By 2024, these specialties remained the largest groups, each exceeding 2.5 specialists per 10,000 population. Notably, the largest change was observed in the number of internists between 2020 and 2021, suggesting that this specialty was particularly affected by the methodological change associated with more detailed reporting.

This same tendency was observed when analyzing the density of healthcare specialists per 1 square kilometer (Table 2). This indicates that the spatial density of healthcare specialists increased both overall and across all analyzed specialties throughout the study period, reflecting improved availability relative to the country’s surface area. In each sub-period—2018–2020 and 2021–2024—specialty-specific densities either remained stable or increased, suggesting a generally upward trend in spatial availability.

In both periods, the largest gains in specialists per 1 square kilometer were observed among oncologists, psychiatrists, and cardiologists. Additionally, in the 2021–2024 period, noticeable increases (of 6% or more) were also recorded among family medicine physicians and radiologists.

When examining individual specialties, in 2020 (before the change in the counting methodology), the largest groups—defined as those exceeding 0.020 specialists per 1 square kilometer—included surgeons, family medicine physicians, and internists. In 2024, the same three specialties continued to represent the largest groups with their density higher than 0.030 specialists per square kilometer. This shows that the spatial distribution pattern aligns closely with the pattern observed for population-based density; in addition, the relatively highest increase of internist could be observed from 2020 to 2021. However, this finding should be interpreted with caution, as it may reflect the methodological change in the way specialists were counted rather than an actual increase in their number.

In line with the study’s aim, the Gini coefficient was used to measure inequalities in the population- and area-based distribution of healthcare specialist supply. The Gini coefficients for the total number of specialists as well as for individual medical fields ranged from 0.05 to 0.21, indicating a relatively equitable distribution in relation to population size (Table 3). Between 2021 and 2024, equity in the distribution of specialists improved in several medical specialties, as indicated by declining Gini coefficient values in seven of the 18 specialties analyzed. For most of the remaining specialties, the level of inequality remained relatively unchanged, while a slight increase was observed for family medicine and for the overall group of specialists.

In 2024, oncologists, endocrinologists, diabetologists, and cardiologists exhibited the highest levels of inequality in their population-based distribution compared with other medical specialties. In contrast, in 2020, the highest levels of inequality were still observed among oncologists, but a notable pattern was also observed among family medicine physicians and occupational medicine specialists. Between 2018 and 2020, the inequality index remained consistently high at 0.19. This was followed by a marked decline in 2021, after which the index remained relatively stable, with only a slight increase of 0.01 by 2024. However, this change should be interpreted with caution, as a methodological revision in physician reporting was introduced in 2021. Therefore, the observed decrease may reflect more accurate reporting of the actual number of physicians rather than a genuine improvement in their distribution, although it could also partly result from the effects of healthcare policies and workforce interventions implemented during this period. Moreover, the overall distributional patterns across specialties remain similar before and after the methodological revision, suggesting that geographical inequalities in the distribution of medical specialists persist despite improvements in specialist reporting (due to a change of methodology).

The Gini coefficients for healthcare specialists, based on their spatial distribution, ranged from 0.23 to 0.39 (Table 4), indicating potential spatial inequalities. These values are higher than the population-based Gini coefficients, which reflects greater inequality when considering distribution per square kilometer. Importantly, an upward trend in Gini coefficient values was observed between 2021 and 2024, suggesting slight deterioration of equity in the spatial distribution in seven healthcare specialists. The period 2018–2020 is characterized by a mixed pattern, as only four specialties experienced a deterioration in equity. However, higher values recorded in 2021 compared with 2020 indicate that relative to the previous period—calculated using a different methodology—the level of inequality initially appeared higher in 2021 and finally with similar level in 2024. Nevertheless, regardless of the methodological change and the improved reporting of the healthcare workforce, the results suggest that inequalities persist and may be more pronounced than previously observed.

Overall, the results suggest that the area-based distribution of healthcare specialists was less equitable than their population-based distribution. Although spatial inequalities were consistently higher throughout the 2021–2024 period, their level remained relatively stable over time. In 2024, the highest levels of inequality in specialist distribution were observed among cardiologists, oncologists, endocrinologists, occupational medicine and ophthalmologists, with Gini coefficients exceeding 0.36. In 2020, the greatest inequalities were also recorded among cardiologists and oncologists, followed by internists and occupational medicine physicians, with Gini coefficients above 0.32.

The Theil index was used to assess overall equity in the distribution of healthcare specialists in Poland and the contribution of the seven Polish macro-regions to regional inequalities. The results are presented in Table 5. These findings reveal not only general trends in geographical inequality but also pronounce differences across healthcare specialties. For the total pool of specialists, there was a gradual increase in inequality, with the Theil index rising from 0.010 in 2018 to 0.011 in 2020 and from 0.013 in 2021 to 0.014 in 2024, indicating modest growth in regional disparities in distribution of healthcare specialists. The decomposition of the index demonstrates a substantial shift in the structure of inequality sources over time. The share of the within-macro-region component decreased from approximately 57–59% in 2018–2020 to 37.79% in 2024, which means the contribution of inequalities between macro-regions increased to 62.21% in 2024.

The decomposition of the Theil index reveals pronounced differences in the sources of inequality across medical specialties. In several fields—most prominently cardiology, oncology, internist, psychiatry, lung diseases, neurology, and endocrinology specialties—disparities were consistently driven by differences between macro-regions, indicating that the macro-regional location within Poland played a decisive role in shaping supply of healthcare specialists. In contrast, specialties such as dermatology and generally otolaryngology (with an exception in 2023) exhibited patterns in which within-macro-region variation had a stronger influence. The remaining specialties (eight of them) demonstrated a gradual shift over time, transitioning from primarily within-region inequality record in 2018–2020 toward patterns increasingly dominated by disparities between macro-regions in the period 2021–2024. This indicates and confirms an evolving inequality structure in which macro-regional divergence has become more prominent. A methodological change introduced in 2021 likely improved the accuracy of physician counts, providing a more reliable representation of the actual number of specialists. Consequently, comparisons before and after 2021 should be interpreted with caution, although the overall pattern of inequalities remained unchanged, which implies the existence of geographical inequities.

Regardless of the change in methodology in 2021, the direction of change is notable: when each sub-period is considered on its own—and when the full span is viewed cumulatively—there is a worsening of equity in 2018–2020 and then again in 2021–2024; when considering specialist in total, however, it is again different for each specialty. In 2018–2020, improvements in equality were observed in several specialties, including dermatology, cardiology, family medicine, neurology, occupational medicine, oncology, ophthalmology, otolaryngology, pediatrics, and radiology. In 2021–2024, the overall pattern shows a general improvement of inequality across almost all specialties (with the exception of anesthesiology). Overall, geographic inequality in 2024 was lower than in 2020 for most medical specialties. The only exceptions were oncology, pediatrics, anesthesiology, ophthalmology, radiology, and otolaryngology, where inequality levels were higher than in the last year to the previous period. When comparing inequalities across specialties, the highest levels of geographical inequality in 2024 were observed among oncologists, cardiologists, psychiatrists, occupational medicine physicians, diabetologists, and endocrinologists.

These findings collectively suggest that, despite improvements in some specialties, regional disparities have widened, indicating an increasing concentration of specialist resources in selected regions.

Moreover, the macro-regional decomposition of the Theil index reveals clear patterns in the geographic distribution of healthcare specialists in Poland. In 2024, across most specialties, the highest levels of inequality were consistently recorded in the eastern macro-regions, which exhibited the greatest internal dispersion in the distribution of specialists. In contrast, the Masovian macro-region demonstrated equity, with values close to zero for many specialties, indicating highly uniform—though often strongly concentrated—distributions. This may, however, be explained by the fact that this macro-region comprises a single voivodeship. When examining specific medical fields, oncology, pediatrics, psychiatry, dermatology, radiology, and endocrinology each displayed their greatest inequalities within these same high-disparity macro-regions.

## 4. Discussion

This study reveals several key findings. First, the results show that there are inequalities in the geographical distribution of healthcare specialists across Poland. A combined analysis of density measures and Gini coefficients shows that an increase in the number of specialists does not automatically lead to a more equitable distribution of specialists across the country. This was particularly evident for oncologists, cardiologists, and psychiatrists, where marked increases in specialist density were not accompanied by corresponding improvements in the equity of their geographical distribution.

The present findings are in line with previous studies reporting inequalities in the distribution of the medical workforce in Poland. Although Guziak et al. (2025) focused exclusively on primary healthcare physicians, their findings closely align with the present study [46]. Using the Map of Health Needs [60], they demonstrated that, despite improvements in the overall geographical distribution of primary healthcare (PHC) physicians, rural areas continue to experience lower physician availability, indicating that equitable distribution has not yet been achieved [46].

Second, inequity in the geographical distribution of healthcare specialists was greater when assessed in relation to area-based than population-based, pointing to persistent regional differences. This finding is consistent with previous evidence from 2010–2017 (Rój, 2020) [18]. Possible explanations for the consistently higher area-based inequalities can be found in the existing literature. Previous studies have shown that the geographical distribution of healthcare specialists is influenced not only by population needs, but also by structural, economic and institutional factors. Specialists tend to cluster in urban centers and academic hubs with stronger infrastructure and training opportunities, while rural and peripheral regions face persistent shortages due to weaker healthcare system capacity, limited professional development prospects and lower economic attractiveness. These mechanisms may partly explain why disparities in the area-based distribution of healthcare specialists are more pronounced than those arising from population distribution alone [61,62,63,64,65]. However, as these factors were not examined in the present study, further research is needed to confirm their contribution to the observed patterns.

Third, the Theil index results confirm that substantial inequalities in the geographical distribution of healthcare specialists persist in Poland. The findings suggest that these disparities are increasingly driven by differences between macro-regions rather than within-region variation. Compared with the 2010–2017 period (Rój, 2020) [18], between-macro-regional disparities appear to have become more pronounced especially in the period 2018–2024. This pattern may have been influenced, at least in part, by the revision of the NUTS classification in 2018, which increased the number of Polish macro-regions from six to seven and more importantly changed the composition of several macro-regions by reallocating selected voivodeships. Additionally, the geographic distribution of physicians may have been affected by the COVID-19 pandemic. Nevertheless, the findings indicate that regional inequalities remain substantial. In 2024, poorer equity of geographical distribution was comparatively observed for oncologists, cardiologists, occupational medicine physicians, psychiatrists, diabetologists and endocrinologists. Compared with 2017 [18], the distribution remained relatively least equitable for oncologists, cardiologists, family medicine physicians, and occupational medicine physicians.

In addition, the highest levels of inequality across most healthcare specialties were observed in the eastern macro-regions, which were characterized by the greatest internal variation in specialist distribution. This suggests that, despite government initiatives and investments aimed at improving healthcare provision, these areas continue to experience relatively unfavorable conditions and may require targeted and sustained support. Similar findings were reported by Domagała et al. [45], who identified significant disparities in access to physicians across Poland, particularly in the east, where peripheral and depopulating regions were found to have poorer access. These disparities led to marked inequalities in workforce distribution [45,47].

Taken together, the Gini coefficient and Theil index findings suggest that inequalities in the geographical distribution of healthcare specialists occur at multiple levels. While the Gini coefficient suggests disparities between rural and urban areas, the Theil index indicates that differences between macro-regions constitute the main source of inequality. These findings highlight the importance of prioritizing actions aimed at reducing interregional disparities, while also addressing persistent gaps in specialist availability within macro-regions.

Fourth, given that cardiovascular and oncological diseases are the leading causes of death in Poland, accounting for over half of the total disease burden (37% and 27%, respectively, according to the statistical report [49]), the observed increase in the number of cardiologists and oncologists per 10,000 people and per square kilometer represents a positive trend. However, the increase in these specialist numbers was not accompanied by improved equity. Population-based inequality remained largely unchanged, whereas geographical inequality, measured in relation to area, increased. In addition, geographical distribution of both oncologists and cardiologists remains less equitable than for most other specialties, as confirmed by the Theil index. These findings broadly align with those for 2010–2017 [18], during which period both groups of healthcare specialists expanded. However, equity trends differed: oncology improved across both measures, whereas cardiology showed declining population-based equity and no change in area-based distribution [18]. Thus, the 2018–2024 results confirm the ongoing problem in equity of geographical distribution of cardiologists and the comparatively better, though still insufficient, situation of oncologists, especially when analyzing population-based distribution. Of particular concern is the fact that these inequities exist—and in some cases worsen—in the two specialties most closely linked to Poland’s leading causes of death, highlighting the seriousness of these trends for public health.

Fifth, previous research by [18] identified persistent shortages and unequal distribution of family doctors, pediatricians, and internists during 2010–2017, accompanied by declining equity [18]. As these specialties play a central role in the provision of primary healthcare services, their distribution is of particular importance. In contrast, the results for 2018–2024 suggest a different pattern, indicating changes in the distribution of physicians providing primary healthcare services. As, the number of family doctors, pediatricians, and internists increased between 2018 and 2024, reversing the earlier decline in the number of internists. Equity trends varied across specialties. Population-based equity improved for family doctors but deteriorated for pediatricians and, to a lesser extent, internists. In contrast, area-based equity deteriorated for family doctors and pediatricians. The Theil index indicated improved equity in distribution of family doctors and worsening inequality among pediatricians and internists. Overall, despite the growth in the primary healthcare workforce, inequalities in the geographical distribution of these specialists persist, particularly when assessed using the area-based Gini coefficient. Despite the methodological change in how the number of physicians is determined—a shift toward data acquisition methods that provide more accurate information on the medical workforce—existing inequalities persist, indicating that this problem has not been resolved.

Thus, these findings suggest that the initiatives and reforms undertaken by the Ministry of Health to strengthen primary healthcare, including the prioritization of family medicine (2003), pediatrics (2009), and internal medicine (2018), as well as the changes introduced under the 2017 Primary Health Care Act, have not fully addressed inequalities in the geographical distribution of these specialists. Strengthening primary healthcare is particularly important in the context of the introduction of coordinated care and the broader shift toward the “inverse care pyramid”, as these reforms expand the role of family physicians and position primary healthcare as the foundation of the healthcare system.

Also, the inclusion of the COVID-19 pandemic period provides valuable insights into the resilience of the healthcare workforce distribution. Despite substantial changes in healthcare demand and service organization during the pandemic, no clear improvement in the geographical equity of specialist distribution was observed. At the same time, the pandemic did not appear to exacerbate existing geographical inequalities, despite the major pressures placed on the healthcare system. These findings suggest that short-term responses to public health emergencies are unlikely to reduce pre-existing regional inequities unless they are accompanied by sustained workforce policies targeting the spatial distribution of healthcare professionals.

The findings of this study contribute to the theoretical understanding of healthcare specialist equity in several ways. Firstly, they provide a clear conceptual distinction between nominal availability (the number of specialists) and effective availability (their equitable, need-consistent spatial distribution). The results show that increases in headcount do not automatically translate into improved equity, highlighting the need to consider healthcare specialists adequacy in terms other than simple numerical supply, as well as the need for a holistic approach to healthcare workforce planning. Secondly, the study introduces an integrated analytical framework combining population- and area-based equity measures with the Gini coefficient and Theil index (including decomposition). This framework not only allows for the measurement of inequity, but also the identification of its structural source, i.e., whether inequalities predominantly arise between or within regions, which connects inequality metrics directly to actionable policy levers. Thirdly, this study situates Poland’s patterns within the broader international literature. Evidence from multiple countries shows that the pandemic widened pre-existing geographic and socioeconomic disparities in access to specialist care as for example [66,67,68]. A recent analysis of 16 countries similarly documents significant income-related disparities in access to general practitioner (GP), surgical, and digital services [69]. The present findings align with these international patterns, demonstrating significant geographic disparities and substantial dominance of high-burden specialties between regions. These results reinforce the conclusion that structural inequalities persist even when the workforce grows, unless resource allocation directly addresses spatial disadvantage.

Thus, beyond its theoretical contributions, this study also has important practical implications.

### 4.1. Regional Workforce Planning and Targeted Policy Interventions for Allocation of Healthcare Human Resources

These findings suggest that reducing geographical inequalities in the distribution of healthcare specialists requires not only increasing the overall supply of specialists but also implementing regionally tailored workforce planning and targeted policy interventions. Such an approach may improve the allocation of healthcare human resources, enhance access to specialist services in underserved regions, and contribute to a more equitable distribution of specialist care across Poland.

Therefore, workforce planning could place greater emphasis on regional demographic, epidemiological, and geographical characteristics when determining specialist training capacity, residency allocation, and workforce priorities. Incorporating population- and area-based equity indicators, such as the Gini and Theil indices, into routine monitoring may facilitate the early identification of regional imbalances and support evidence-informed workforce planning.

The findings further suggest that recent national initiatives, including increased medical school admissions and residency positions, should be complemented by region-sensitive workforce policies. Future planning could therefore expand the parameters currently applied in BASW [70] by placing greater emphasis on documented regional shortages and projected healthcare needs.

To address geographical inequities in the distribution of healthcare specialists, future workforce policies should also place greater emphasis on the determinants of specialty choice. Given that medical education is a key driver of the future healthcare workforce, educational and training policies should be aligned with anticipated population health needs and regional workforce requirements, thereby contributing to a more equitable distribution of specialists.

International evidence suggests that reducing regional workforce inequities requires a combination of financial and non-financial measures tailored to national and regional contexts [46,71]. In Poland, physicians’ specialty choice is influenced by labor market conditions, professional values, and social factors [72,73,74], indicating that workforce policies should take these determinants into account while improving the attractiveness of practicing in underserved regions. Given the central role of medical education in shaping the future specialist workforce, educational and training policies should also better align postgraduate training capacity with projected population health needs and regional workforce demands. Regular monitoring of medical students’ specialty choices should also be strengthened to ensure that incentive mechanisms remain aligned with evolving expectations and to inform reforms aimed at improving the attractiveness of primary care and other specialties facing workforce shortages.

The findings of this study further suggest that such policies should be specialty-specific, as geographical inequities differ considerably across specialist groups and according to the measure of accessibility used. Particular attention should be given to specialties characterized by both persistent geographical inequities and a high disease burden, especially cardiology and oncology, as well as to regions consistently experiencing limited access to specialist care.

To address these challenges, broad national initiatives—such as increasing medical school admissions or expanding residency training—must be complemented by region-specific workforce instruments. Where inequalities are primarily observed between macro-regions, allocation strategies should focus on directing new capacity to underserved areas through mechanisms such as specialist quotas, location-weighted residency places linked to post-training deployment, and dedicated regional recruitment schemes.

In this context, stronger and more flexible contracting mechanisms by the National Health Fund (NHF) are also essential. NHF contracts should be explicitly adjusted to account for documented regional shortages, and multi-site service models could be expanded to extend specialist care to peripheral and rural regions. These measures would allow workforce planning objectives to be translated more effectively into actual service availability.

Health policies interventions should also place greater emphasis on retention and redistribution of healthcare specialists. Targeted incentive packages—including relocation grants, bonded stipends, housing or childcare support, and structured regional career pathways—may be necessary to attract and retain specialists in the most underserved areas. Such instruments are particularly important given that current shortages and spatial imbalances persist despite recent reforms and the objectives of national programs such as the National Oncology Strategy (2020–2030) and the National Program for Cardiovascular Diseases (2022–2032).

Continuous evaluation of Ministry of Health initiatives is therefore essential, especially to assess whether implemented measures improve physician availability in rural and deficit regions or unintentionally contribute to widening inequalities. The findings of this study may also serve as a valuable basis for cross-country comparisons and may support other countries in adapting effective workforce policies and organizational solutions to address regional inequalities in the distribution of healthcare professionals.

### 4.2. Effective Policies for Fairer Distribution of Specialist Care Across the Country

The findings of this study further indicate that effective healthcare workforce policies should be evaluated not only by the number of specialists entering the healthcare system, but also by their ability to improve the equity of geographical distribution. Increasing specialist supply without reducing regional disparities may lead to further concentration of healthcare professionals in already well-served areas, limiting the effectiveness of public investment and failing to improve access to specialist healthcare for populations living in underserved regions. Consequently, equity indicators should become an integral component of workforce policy evaluation, complementing traditional measures based solely on workforce numbers.

From a broader health policy perspective, promoting a more equitable geographical distribution of healthcare specialists represents not only an organizational objective but also an important strategy for improving health system performance. Better alignment between workforce allocation and regional healthcare needs may contribute to more timely access to specialist services, reduce avoidable regional differences in health outcomes, and strengthen the resilience of the healthcare system to future demographic and epidemiological challenges. Therefore, effective policies should combine long-term planning, continuous monitoring of geographical equity, and regular evaluation of implemented interventions to ensure that increases in workforce capacity translate into meaningful improvements in access to care.

Finally, although this study focused on the geographical distribution of healthcare specialists, future workforce policies could also be supported by broader public health initiatives. Strengthening prevention, early detection, and risk-based screening programs may help reduce the future demand for highly specialized services, while region-sensitive workforce planning, targeted recruitment and retention strategies, and NHF contracting that reflects documented regional shortages may contribute to a more balanced distribution of healthcare specialists.

#### Limitation of the Study and Future Research

This study has some limitations, the main one being the change in methodology in 2021, which makes comparisons between earlier and later years somewhat more challenging and requires a cautious interpretation of the findings. Additionally, the use of large macro-regions may hide smaller, local shortages. Moreover, the database lacked information on the specific skills or qualifications of healthcare specialists, meaning the analysis could only address staffing numbers rather than competency profiles. As there were no detailed data on the actual use of healthcare services, it was not possible to compare workforce availability with real patient demand.

Future research should aim to link the distribution of healthcare specialists with actual service use and healthcare outcomes, such as waiting times and avoidable hospitalizations, to reflect real access to healthcare. It should also examine the underlying drivers of these macro-regional disparities, including funding mechanisms, differences in training capacity, and the relative attractiveness of specific regions for specialists. Future work should explore staff mobility and retention patterns, as well as patient flows between regions. More comprehensive datasets should be built that link workforce characteristics with service utilization and health outcomes. As the choice of medical specialty by young doctors is essential for maintaining an adequate healthcare workforce and ensuring the sustainability of the system, broad and comprehensive research into the determinants of specialty selection is crucial when considering larger and more diverse physician populations.

## 5. Conclusions

Building on earlier work by [18] this study examined the geographical distribution of healthcare specialists in Poland during 2018–2024. The findings suggest that regional inequities persist and, for several specialties, have increased over time. Area-based inequality was consistently higher than population-based inequality, highlighting persistent spatial imbalances in the availability of specialist care. Although the overall number of specialists increased, this did not necessarily translate into a more equitable distribution. This was particularly evident in cardiology and oncology, where substantial geographical inequities persisted despite growth in specialist numbers. The Theil decomposition further showed that differences between macro-regions constitute an increasingly important source of inequality across several specialties.

These findings may support evidence-based workforce allocation decisions. They suggest that increasing the overall supply of healthcare specialists alone is insufficient to reduce regional inequities. Instead, workforce planning should adopt a more region- and specialty-sensitive approach that considers demographic and epidemiological trends, geographical accessibility, and regional healthcare needs when determining specialist training capacity, residency allocation, and workforce priorities. Since the magnitude of geographical inequities varied considerably across specialist groups, policy interventions should be tailored to the specific needs of individual specialties, particularly those characterized by both persistent inequities and a high disease burden. Regular monitoring using both population- and area-based indicators may support the early identification of regional imbalances and enable more evidence-informed allocation of healthcare human resources.

Importantly, the inclusion of the COVID-19 pandemic period indicates that temporary crisis response measures alone are insufficient to improve the geographical equity of specialist distribution, reinforcing the need for sustained, region-sensitive workforce policies. At the same time, the findings suggest that the pandemic did not exacerbate existing geographical inequalities in the distribution of specialists, despite the unprecedented pressures placed on the healthcare system.

Overall, these findings reinforce international evidence that geographical inequities in the healthcare workforce remain a persistent challenge and demonstrate that expanding specialist numbers alone is unlikely to improve equity unless accompanied by regionally tailored workforce planning and targeted policy interventions. In particular, prioritizing specialties and macro-regions with the greatest documented inequalities may improve the allocation of healthcare human resources and contribute to a fairer distribution of specialist care across Poland. The results of this study may also support other countries facing similar challenges in adapting evidence-informed workforce policies to their own healthcare systems.

## Figures and Tables

**Table 1:** Density of healthcare specialists (per 10,000 population) in Poland in the years from 2018 to 2024.

Year	2018	2019	2020	2021	2022	2023	2024	Change from 2018 to 2020 *	Change from 2021 to 2024 *	Change from 2018 to 2024 *
specialist in general	15.73	15.87	16.26	22.81	23.15	24.15	24.19	3.40%	6.05%	53.80%
anesthesiologist	1.00	1.01	1.05	1.41	1.42	1.49	1.50	4.62%	6.66%	49.37%
cardiologist	0.78	0.80	0.83	1.29	1.33	1.39	1.41	6.63%	9.43%	80.63%
dermatologist	0.22	0.23	0.23	0.48	0.48	0.50	0.50	5.65%	5.70%	126.26%
diabetologist	n.a. ^1^	n.a. ^1^	n.a. ^1^	0.39	0.40	0.41	0.40	n.a. ^1^	4.98%	n.a. ^1^
endocrinologist	n.a. ^1^	n.a. ^1^	n.a. ^1^	0.41	0.42	0.44	0.44	n.a. ^1^	7.77%	n.a. ^1^
family medicine	1.76	1.77	1.78	2.65	2.78	2.86	2.88	1.27%	8.46%	63.74%
internist	1.64	1.64	1.68	4.71	4.75	4.91	4.84	1.95%	2.78%	194.17%
lung diseases	0.33	0.33	0.33	0.62	0.62	0.65	0.62	−1.36%	−0.39%	85.46%
neurologist	0.62	0.63	0.65	0.99	1.00	1.05	1.05	3.69%	6.15%	68.73%
obstetrics and gynecology	0.95	0.93	0.96	1.59	1.59	1.64	1.64	0.52%	3.58%	72.55%
occupational medicine	0.21	0.21	0.21	n.a. ^1^	0.40	0.41	0.40	1.13%	−0.22%	89.12%
oncologist	0.21	0.22	0.22	0.33	0.35	0.36	0.37	7.85%	11.45%	79.45%
ophthalmologist	0.51	0.52	0.52	0.91	0.92	0.96	0.97	2.59%	6.48%	90.31%
otolaryngology	0.35	0.35	0.35	0.55	0.55	0.58	0.58	0.89%	6.07%	68.43%
pediatrician	0.93	0.92	0.92	1.87	1.90	1.98	1.98	−0.87%	5.97%	112.01%
psychiatrist	0.62	0.64	0.65	0.99	1.02	1.06	1.09	5.42%	11.11%	77.71%
radiologist	0.63	0.62	0.64	1.02	1.05	1.12	1.16	2.76%	13.51%	85.76%
surgeon	2.22	2.27	2.39	3.37	3.37	3.50	3.53	7.69%	4.84%	58.96%

Source: Own calculations; * the calculated change took into account more places (numbers) after the full-stop. ^1^ n.a. = not available.

**Table 2:** Density of healthcare specialists (per 1 square km) in Poland in the years from 2018 to 2024.

Year	2018	2019	2020	2021	2022	2023	2024	Change from 2018 to 2020 *	Change from 2021 to 2024 *	Change from 2018 to 2024 *
specialist in general	0.193	0.195	0.200	0.279	0.281	0.291	0.290	3.26%	3.90%	50.02%
anesthesiologist	0.012	0.012	0.013	0.017	0.017	0.018	0.018	4.48%	4.49%	45.69%
cardiologist	0.010	0.010	0.010	0.016	0.016	0.017	0.017	6.48%	7.21%	76.18%
dermatologist	0.003	0.003	0.003	0.006	0.006	0.006	0.006	5.51%	3.55%	120.70%
diabetology	n.a. ^1^	n.a. ^1^	n.a. ^1^	0.005	0.005	0.005	0.005	n.a. ^1^	2.85%	n.a. ^1^
endocrinology	n.a. ^1^	n.a. ^1^	n.a. ^1^	0.005	0.005	0.005	0.005	n.a. ^1^	5.58%	n.a. ^1^
family medicine	0.022	0.022	0.022	0.032	0.034	0.034	0.034	1.14%	6.26%	59.71%
internist	0.020	0.020	0.021	0.058	0.058	0.059	0.058	1.82%	0.70%	186.94%
lung diseases	0.004	0.004	0.004	0.008	0.008	0.008	0.007	−1.49%	−2.41%	80.90%
neurologist	0.008	0.08	0.008	0.012	0.012	0.013	0.013	3.55%	4.00%	64.58%
obstetrics and gynecology	0.012	0.011	0.012	0.019	0.019	0.020	0.020	0.38%	1.48%	68.30%
occupational medicine	0.003	0.003	0.003	n.a. ^1^	0.005	0.005	0.005	0.99%	−1.31%	84.47%
oncologist	0.003	0.003	0.003	0.004	0.004	0.004	0.004	7.71%	9.19%	75.03%
ophthalmologist	0.006	0.006	0.006	0.011	0.011	0.012	0.012	2.45%	4.33%	85.63%
otolaryngology	0.004	0.004	0.004	0.007	0.007	0.007	0.007	0.75%	3.92%	64.29%
pediatrician	0.011	0.011	0.011	0.023	0.023	0.024	0.024	−1.01%	3.82%	106.79%
psychiatrist	0.008	0.008	0.008	0.012	0.012	0.013	0.013	5.28%	8.85%	73.34%
radiologist	0.008	0.008	0.008	0.013	0.013	0.014	0.014	2.62%	11.20%	81.19%
surgeon	0.027	0.028	0.029	0.041	0.041	0.042	0.042	7.55%	2.72%	55.05%

Source: Own calculations. * the calculated change took into account more places (numbers) after the full-stop. ^1^ n.a. = data not available.

**Table 3:** Gini coefficients of population distribution of healthcare specialists in the years from 2018 to 2024.

Year	2018	2019	2020	2021	2022	2023	2024
specialist in general	0.07	0.05	0.05	0.09	0.09	0.11	0.10
anesthesiologist	0.06	0.08	0.09	0.10	0.08	0.10	0.10
cardiologist	0.14	0.16	0.14	0.15	0.14	0.16	0.15
dermatologist	0.16	0.14	0.14	0.14	0.14	0.13	0.13
diabetology	n.a. ^1^	n.a. ^1^	n.a. ^1^	0.16	0.16	0.17	0.15
endocrinology	n.a. ^1^	n.a. ^1^	n.a. ^1^	0.18	0.18	0.19	0.17
family medicine	0.19	0.19	0.19	0.12	0.13	0.14	0.13
internist	0.12	0.11	0.10	0.13	0.12	0.13	0.12
lung diseases	0.09	0.09	0.10	0.10	0.09	0.10	0.10
neurologist	0.10	0.12	0.10	0.11	0.11	0.12	0.11
obstetrics and gynecology	0.10	0.11	0.11	0.08	0.08	0.08	0.08
occupational medicine	0.21	0.15	0.17	n.a. ^1^	0.15	0.15	0.14
oncologist	0.16	0.18	0.16	0.17	0.12	0.21	0.15
ophthalmologist	0.14	0.12	0.11	0.13	0.12	0.13	0.12
otolaryngology	0.10	0.09	0.09	0.12	0.11	0.12	0.10
pediatrician	0.11	0.10	0.10	0.13	0.12	0.14	0.13
psychiatrist	0.12	0.11	0.12	0.13	0.14	0.15	0.14
radiologist	0.16	0.13	0.12	0.14	0.13	0.14	0.14
surgeon	0.07	0.07	0.08	0.08	0.08	0.08	0.08

Source: Own calculations. ^1^ n.a. = data not available.

**Table 4:** Gini coefficients of geographic distribution of healthcare specialists in the years from 2018 to 2024.

Year	2018	2019	2020	2021	2022	2023	2024
specialist in general	0.29	0.28	0.28	0.32	0.32	0.32	0.32
anesthesiologist	0.28	0.29	0.29	0.33	0.33	0.34	0.34
cardiologist	0.34	0.37	0.37	0.38	0.38	0.39	0.38
dermatologist	0.31	0.27	0.30	0.32	0.32	0.32	0.32
diabetology	n.a. ^1^	n.a. ^1^	n.a. ^1^	0.33	0.33	0.34	0.33
endocrinology	n.a. ^1^	n.a. ^1^	n.a. ^1^	0.35	0.36	0.37	0.36
family medicine	0.24	0.23	0.23	0.24	0.24	0.25	0.25
internist	0.35	0.34	0.34	0.36	0.35	0.36	0.35
lung diseases	0.30	0.30	0.29	0.30	0.30	0.31	0.31
neurologist	0.31	0.30	0.30	0.32	0.32	0.33	0.32
obstetrics and gynecology	0.26	0.26	0.27	0.31	0.31	0.31	0.31
occupational medicine	0.39	0.34	0.32	n.a. ^1^	0.37	0.37	0.36
oncologist	0.33	0.37	0.34	0.36	0.35	0.39	0.36
ophthalmologist	0.34	0.31	0.31	0.35	0.36	0.36	0.36
otolaryngology	0.30	0.29	0.27	0.32	0.32	0.33	0.32
pediatrician	0.31	0.29	0.31	0.33	0.33	0.34	0.34
psychiatrist	0.33	0.31	0.31	0.33	0.34	0.35	0.34
radiologist	0.33	0.31	0.30	0.32	0.31	0.32	0.32
surgeon	0.28	0.28	0.27	0.32	0.32	0.32	0.32

Source: Own calculations. ^1^ n.a. = data not available.

**Table 5:** Theil index of specialist distribution in the years from 2018–2024.

		Theil Index									
Types of Healthcare Human Resources	Year	Poland	Central	South West	North West	South	North	East	Maso-vian	Within Groups	Among Groups
specialists in general	2018	0.010	0.000	0.001	0.029	0.000	0.004	0.001	0.000	57.47%	42.53%
	2019	0.013	0.000	0.001	0.036	0.000	0.008	0.001	0.000	57.11%	42.89%
	2020	0.011	0.000	0.001	0.030	0.000	0.007	0.002	0.000	58.96%	41.04%
	2021	0.013	0.008	0.012	0.004	0.000	0.005	0.014	0.000	39.82%	60.18%
	2022	0.012	0.007	0.011	0.004	0.000	0.004	0.015	0.000	42.74%	57.26%
	2023	0.018	0.007	0.013	0.004	0.000	0.004	0.015	0.000	30.61%	69.39%
	2024	0.014	0.007	0.013	0.004	0.000	0.004	0.013	0.000	37.79%	62.21%
anesthesiologist	2018	0.006	0.001	0.000	0.003	0.011	0.004	0.010	0.000	81.21%	18.79%
	2019	0.013	0.000	0.001	0.000	0.000	0.000	0.011	0.000	70.03%	29.97%
	2020	0.011	0.000	0.000	0.000	0.000	0.000	0.018	0.000	70.56%	29.44%
	2021	0.013	0.011	0.008	0.000	0.000	0.000	0.026	0.000	48.23%	51.77%
	2022	0.012	0.008	0.002	0.000	0.000	0.000	0.026	0.000	50.87%	49.13%
	2023	0.018	0.011	0.001	0.000	0.000	0.000	0.024	0.000	40.82%	59.18%
	2024	0.014	0.015	0.006	0.000	0.001	0.002	0.025	0.000	52.68%	47.32%
cardiologist	2018	0.039	0.003	0.000	0.017	0.001	0.018	0.002	0.000	16.40%	83.60%
	2019	0.050	0.000	0.000	0.037	0.001	0.033	0.000	0.000	22.26%	77.74%
	2020	0.037	0.000	0.000	0.009	0.000	0.028	0.002	0.000	16.13%	83.87%
	2021	0.037	0.003	0.016	0.001	0.000	0.012	0.010	0.000	14.77%	85.23%
	2022	0.034	0.001	0.018	0.001	0.000	0.010	0.011	0.000	15.09%	84.91%
	2023	0.041	0.001	0.022	0.005	0.000	0.013	0.010	0.000	15.65%	84.35%
	2024	0.034	0.002	0.017	0.004	0.000	0.011	0.009	0.000	16.19%	83.81%
dermatologist	2018	0.043	0.024	0.000	0.151	0.023	0.006	0.012	0.000	79.82%	20.18%
	2019	0.036	0.023	0.003	0.105	0.016	0.007	0.009	0.000	68.92%	31.08%
	2020	0.039	0.008	0.010	0.124	0.033	0.008	0.005	0.000	78.54%	21.46%
	2021	0.026	0.050	0.021	0.044	0.000	0.011	0.013	0.000	66.77%	33.23%
	2022	0.023	0.039	0.028	0.034	0.000	0.020	0.010	0.000	73.18%	26.82%
	2023	0.025	0.026	0.017	0.032	0.000	0.015	0.008	0.000	51.71%	48.29%
	2024	0.020	0.030	0.027	0.028	0.000	0.027	0.003	0.000	73.13%	26.87%
family medicine	2018	0.066	0.000	0.001	0.160	0.023	0.001	0.020	0.000	51.41%	48.59%
	2019	0.064	0.000	0.001	0.161	0.019	0.019	0.016	0.000	55.33%	44.67%
	2020	0.062	0.000	0.004	0.173	0.020	0.007	0.016	0.000	57.43%	42.57%
	2021	0.021	0.000	0.016	0.007	0.009	0.000	0.029	0.000	41.49%	58.51%
	2022	0.024	0.000	0.013	0.005	0.006	0.001	0.036	0.000	35.85%	64.15%
	2023	0.025	0.000	0.019	0.005	0.006	0.001	0.033	0.000	34.40%	65.60%
	2024	0.021	0.000	0.016	0.004	0.005	0.001	0.032	0.000	36.13%	63.87%
internist	2018	0.021	0.008	0.005	0.016	0.004	0.003	0.000	0.000	25.32%	74.68%
	2019	0.022	0.009	0.001	0.024	0.002	0.002	0.004	0.000	28.41%	71.59%
	2020	0.021	0.009	0.003	0.026	0.001	0.000	0.005	0.000	29.45%	70.55%
	2021	0.023	0.007	0.018	0.015	0.000	0.011	0.019	0.000	39.54%	60.46%
	2022	0.021	0.007	0.015	0.013	0.000	0.007	0.019	0.000	38.33%	61.67%
	2023	0.028	0.006	0.019	0.014	0.000	0.005	0.021	0.000	30.14%	69.86%
	2024	0.020	0.006	0.017	0.013	0.000	0.006	0.018	0.000	37.42%	62.58%
lung disease	2018	0.012	0.000	0.000	0.018	0.002	0.005	0.007	0.000	40.94%	59.06%
	2019	0.015	0.000	0.001	0.014	0.002	0.012	0.004	0.000	35.11%	64.89%
	2020	0.017	0.000	0.002	0.012	0.008	0.014	0.009	0.000	43.35%	56.65%
	2021	0.014	0.001	0.002	0.004	0.012	0.002	0.012	0.000	38.02%	61.98%
	2022	0.011	0.000	0.002	0.004	0.010	0.001	0.008	0.000	37.47%	62.53%
	2023	0.016	0.000	0.000	0.005	0.011	0.003	0.006	0.000	27.61%	72.39%
	2024	0.012	0.000	0.000	0.005	0.010	0.001	0.008	0.000	35.94%	64.06%
obstetrics and gynecology	2018	0.017	0.000	0.028	0.058	0.010	0.011	0.002	0.000	94.03%	5.97%
	2019	0.017	0.004	0.031	0.042	0.010	0.019	0.001	0.000	88.32%	11.68%
	2020	0.018	0.006	0.033	0.036	0.013	0.024	0.002	0.000	90.79%	9.21%
	2021	0.011	0.019	0.003	0.002	0.000	0.001	0.011	0.000	38.80%	61.20%
	2022	0.009	0.018	0.003	0.003	0.001	0.000	0.011	0.000	45.86%	54.14%
	2023	0.012	0.012	0.001	0.003	0.002	0.001	0.011	0.000	29.35%	70.65%
	2024	0.009	0.014	0.003	0.003	0.001	0.001	0.009	0.000	41.40%	58.60%
neurologist	2018	0.019	0.005	0.001	0.011	0.002	0.000	0.016	0.000	27.32%	72.68%
	2019	0.027	0.005	0.001	0.016	0.002	0.009	0.014	0.000	26.37%	73.63%
	2020	0.017	0.005	0.001	0.014	0.004	0.006	0.013	0.000	38.46%	61.54%
	2021	0.018	0.000	0.007	0.005	0.010	0.003	0.008	0.000	29.60%	70.40%
	2022	0.016	0.000	0.005	0.005	0.009	0.002	0.009	0.000	28.64%	71.36%
	2023	0.020	0.001	0.008	0.008	0.010	0.001	0.006	0.000	25.24%	74.76%
	2024	0.016	0.001	0.007	0.005	0.009	0.001	0.007	0.000	28.51%	71.69%
occupational medicine	2018	0.078	0.019	0.025	0.107	0.032	0.119	0.041	0.000	66.79%	33.21%
	2019	0.046	0.018	0.002	0.101	0.017	0.017	0.029	0.000	61.68%	38.32%
	2020	0.043	0.025	0.009	0.117	0.028	0.010	0.021	0.000	74.35%	25.65%
	2021	n.a. ^1^	n.a. ^1^	n.a. ^1^	n.a. ^1^	n.a. ^1^	n.a. ^1^	n.a. ^1^	n.a. ^1^	n.a. ^1^	n.a. ^1^
	2022	0.033	0.001	0.004	0.004	0.029	0.010	0.016	0.000	32.50%	67.50%
	2023	0.038	0.001	0.002	0.007	0.035	0.008	0.010	0.000	29.37%	70.63%
	2024	0.027	0.002	0.001	0.003	0.026	0.009	0.017	0.000	35.63%	64.37%
oncologist	2018	0.042	0.028	0.037	0.003	0.000	0.008	0.038	0.000	32.62%	67.38%
	2019	0.047	0.013	0.104	0.002	0.007	0.012	0.040	0.000	44.45%	55.55%
	2020	0.039	0.032	0.119	0.000	0.000	0.004	0.060	0.000	62.62%	37.38%
	2021	0.053	0.023	0.129	0.011	0.004	0.009	0.025	0.000	43.18%	56.82%
	2022	0.040	0.008	0.082	0.005	0.004	0.010	0.030	0.000	40.37%	59.63%
	2023	0.101	0.007	0.077	0.011	0.006	0.022	0.028	0.000	18.72%	81.28%
	2024	0.040	0.006	0.110	0.008	0.007	0.008	0.024	0.000	47.72%	52.28%
ophthalmologist	2018	0.034	0.001	0.029	0.117	0.011	0.019	0.012	0.000	84.06%	15.94%
	2019	0.036	0.001	0.012	0.136	0.008	0.010	0.006	0.000	75.60%	24.40%
	2020	0.021	0.002	0.012	0.050	0.018	0.006	0.004	0.000	71.17%	28.83%
	2021	0.026	0.032	0.035	0.004	0.002	0.013	0.017	0.000	46.11%	53.89%
	2022	0.022	0.023	0.027	0.005	0.005	0.008	0.019	0.000	47.47%	52.53%
	2023	0.028	0.026	0.028	0.006	0.005	0.010	0.017	0.000	39.69%	60.31%
	2024	0.022	0.019	0.033	0.007	0.005	0.006	0.019	0.000	48.41%	51.59%
otolaryngology	2018	0.012	0.013	0.000	0.027	0.003	0.014	0.010	0.000	82.73%	17.27%
	2019	0.011	0.014	0.001	0.011	0.003	0.028	0.004	0.000	78.51%	21.49%
	2020	0.009	0.004	0.000	0.021	0.002	0.017	0.003	0.000	78.23%	21.77%
	2021	0.018	0.038	0.013	0.021	0.001	0.005	0.026	0.000	72.91%	27.09%
	2022	0.016	0.025	0.008	0.018	0.001	0.003	0.026	0.000	65.92%	34.08%
	2023	0.023	0.024	0.011	0.020	0.004	0.001	0.018	0.000	43.34%	56.66%
	2024	0.014	0.018	0.012	0.016	0.003	0.002	0.021	0.000	62.73%	37.27%
pediatrician	2018	0.022	0.029	0.001	0.024	0.004	0.006	0.025	0.000	54.76%	45.24%
	2019	0.017	0.011	0.000	0.022	0.005	0.006	0.016	0.000	51.24%	48.76%
	2020	0.016	0.010	0.000	0.021	0.002	0.007	0.015	0.000	49.60%	50.40%
	2021	0.022	0.030	0.028	0.020	0.001	0.007	0.014	0.000	56.11%	43.89%
	2022	0.019	0.034	0.024	0.017	0.000	0.005	0.016	0.000	59.58%	40.42%
	2023	0.027	0.035	0.023	0.019	0.001	0.009	0.019	0.000	47.15%	52.85%
	2024	0.021	0.033	0.021	0.021	0.001	0.010	0.017	0.000	59.47%	40.53%
psychiatrist	2018	0.036	0.002	0.001	0.070	0.001	0.024	0.011	0.000	46.83%	53.17%
	2019	0.039	0.003	0.000	0.094	0.001	0.004	0.018	0.000	48.49%	51.51%
	2020	0.038	0.003	0.000	0.065	0.000	0.003	0.023	0.000	38.50%	61.50%
	2021	0.026	0.012	0.012	0.002	0.004	0.017	0.021	0.000	35.80%	64.20%
	2022	0.026	0.016	0.017	0.005	0.002	0.017	0.021	0.000	39.08%	60.92%
	2023	0.036	0.015	0.022	0.005	0.004	0.023	0.023	0.000	33.19%	66.81%
	2024	0.026	0.022	0.023	0.006	0.002	0.022	0.019	0.000	44.85%	55.15%
surgeon	2018	0.006	0.001	0.000	0.015	0.002	0.008	0.004	0.000	75.69%	24.31%
	2019	0.009	0.000	0.000	0.019	0.001	0.022	0.001	0.000	72.11%	27.89%
	2020	0.009	0.000	0.000	0.015	0.001	0.019	0.001	0.000	67.59%	32.41%
	2021	0.010	0.004	0.005	0.005	0.001	0.000	0.011	0.000	36.32%	63.68%
	2022	0.009	0.005	0.007	0.005	0.002	0.000	0.013	0.000	45.83%	54.17%
	2023	0.012	0.005	0.009	0.005	0.003	0.000	0.011	0.000	35.95%	64.05%
	2024	0.009	0.006	0.009	0.005	0.003	0.000	0.012	0.000	47.79%	52.21%
radiologist	2018	0.032	0.009	0.127	0.050	0.016	0.022	0.013	0.000	94.88%	5.12%
	2019	0.024	0.015	0.072	0.038	0.017	0.008	0.007	0.000	84.87%	15.13%
	2020	0.019	0.010	0.052	0.033	0.015	0.007	0.011	0.000	89.02%	10.98%
	2021	0.026	0.000	0.021	0.008	0.002	0.049	0.031	0.000	59.24%	40.76%
	2022	0.022	0.001	0.019	0.006	0.003	0.041	0.031	0.000	64.11%	35.89%
	2023	0.028	0.000	0.021	0.007	0.002	0.036	0.034	0.000	49.57%	50.43%
	2024	0.023	0.000	0.020	0.006	0.004	0.034	0.028	0.000	56.88%	43.12%
diabetology	2021	0.031	0.000	0.002	0.022	0.000	0.003	0.066	0.000	50.78%	49.22%
	2022	0.030	0.000	0.004	0.026	0.001	0.001	0.062	0.000	52.37%	47.63%
	2023	0.050	0.000	0.003	0.018	0.001	0.000	0.088	0.000	35.72%	64.28%
	2024	0.027	0.021	0.003	0.021	0.003	0.003	0.047	0.000	48.93%	51.07%
endocrinology	2021	0.059	0.004	0.010	0.022	0.011	0.032	0.018	0.000	24.77%	75.23%
	2022	0.050	0.007	0.013	0.030	0.004	0.026	0.024	0.000	30.39%	69.61%
	2023	0.059	0.008	0.011	0.042	0.006	0.031	0.030	0.000	32.16%	67.84%
	2024	0.046	0.006	0.006	0.049	0.003	0.034	0.014	0.000	34.47%	65.53%

Source: Own calculations. ^1^ n.a. = data not available.

## Data Availability

The data used in this study are publicly available from Statistics Poland (GUS). For the convenience of readers and to facilitate reproducibility, the datasets used in the analyses are also provided in the Supplementary Materials.

## Supplementary Materials

The following supporting information can be downloaded at: https://www.mdpi.com/article/10.3390/healthcare14152427/s1, Table S1: Structure of healthcare specialists from 2018 to 2024.
