# Supplementary material for: Geographical Inequalities in the Distribution of Healthcare Specialists in Poland
Source: Healthcare (Basel). 2026 Aug 6;14(15):2427. doi: 10.3390/healthcare14152427 (PMC13463840; doi:10.3390/healthcare14152427)
Supplement: Supplementary file 1 [file healthcare-14-02427-s001.zip › healthcare-4324152-supplementary.pdf]

Supplementary

Table S1. Structure of healthcare specialists from 2018 to 2024

Year	2018	2019	2020	2021	2022	2023	2024
Anesthesiology and intensive therapy	7.74%	7.69%	7.83%	5.97%	5.82%	5.89%	5.91%
Surgery	17.10%	17.33%	17.81%	14.29%	13.84%	13.85%	13.91%
Pulmonary diseases	2.56%	2.55%	2.44%	2.63%	2.56%	2.55%	2.43%
Internal diseases	12.67%	12.54%	12.50%	19.99%	19.51%	19.39%	19.08%
Dermatology *and venereology*	1.71%	1.79%	1.75%	2.02%	1.97%	1.97%	1.98%
Cardiology	6.02%	6.13%	6.21%	5.48%	5.47%	5.50%	5.57%
General Practitioners (family)	13.53%	13.50%	13.26%	11.26%	11.41%	11.30%	11.34%
Occupational health	1.61%	1.63%	1.58%	0.00%	1.63%	1.62%	1.56%
Neurology)	4.80%	4.79%	4.81%	4.20%	4.11%	4.14%	4.14%
Ophthalmology	3.92%	3.95%	3.89%	3.87%	3.78%	3.78%	3.82%
Oncology	1.59%	1.70%	1.65%	1.41%	1.42%	1.43%	1.46%
Otolaryngology	2.66%	2.68%	2.60%	2.33%	2.26%	2.29%	2.30%
Paediatrics	7.19%	7.03%	6.89%	7.92%	7.81%	7.82%	7.80%
Obstetrics and gynaecology	7.34%	7.13%	7.14%	6.74%	6.55%	6.47%	6.48%
Psychiatry	4.75%	4.85%	4.84%	4.18%	4.18%	4.19%	4.32%
Radiodiagnostics	4.82%	4.73%	4.79%	4.35%	4.32%	4.44%	4.58%
Diabetologii	0.00%	0.00%	0.00%	1.64%	1.63%	1.62%	1.59%
Endokrynologii	0.00%	0.00%	0.00%	1.74%	1.73%	1.74%	1.74%
